# Treatment of Cataract

**Published:** 1846-12

**Authors:** 


*Treatment of Cataract.*—At the session of the Congress of Italian *savans*, held in Naples last year, Prof. Paliotti recommended as a remedy for cataract the internal use of iodide of potassium, and ammoniacal cauterization on the temples. He declared that even in cases where this treatment did not effect a cure, it rendered the success of the operation more certain. M.M. Quadri and de Horatus were appointed to make experiments with this method and report the result of their observations at the next session.—*Gaz. Médicale.—Ibid.*

